# Supplementary material for: Methylscaper: an R/Shiny app for joint visualization of DNA methylation and nucleosome occupancy in single-molecule and single-cell data
Source: Bioinformatics. 2021 Jun 14;37(24):4857–9. doi: 10.1093/bioinformatics/btab438 (PMC8665741; doi:10.1093/bioinformatics/btab438)
Supplement: btab438_Supplementary_Data [file btab438_supplementary_data.zip › supplement_Revision.docx]

**Supplementary Methods**

1**. Description of methylscaper preprocessing procedure**

Methylscaper allows either single-molecule (e.g., MAPit-BGS) or single-cell (e.g., scNMT-seq) data to be given as input. Single-cell or genome-wide experimental data should be initially processed with the Bismark software tool or other similar alignment programs. Methylscaper expects the input files to have three columns: chromosome, position, and methylation rate. When using Bismark, the output of the bismark_methylation_extractor function using the

--bedGraph option produces a four- and six-column file, which methylscaper will automatically convert to the three-column format either in the subsetSC function of the R package or the preprocessing tab in the Shiny application. Given the size of single-cell files, the subsetSC function will also filter the input to the chromosome level (an example of this is given in the methylscaper package vignette). For single-cell data, if needed, we binarize the methylation rate by designating a rate of 0 as unmethylated and setting any rate greater than 0 to 1 (methylated).

Single-molecule sequence data should be given in the FASTA format, containing the read sequences, along with a reference sequence file in FASTA format. The reference sequence file should be input in the 5’ to 3’ orientation (Watson strand). The alignment is done using the pairwiseAlignment function in the Biostrings R package. We set the alignment penalty to 1 for exact matching and for C-T or G-A conversions. We align each read and its reverse compliment to the Watson and the Crick strands of the reference sequence similar to the BSMAP procedure (Xi and Li, 2009). Nonmatching alignments receive a penalty of -5. The gap opening penalty is -8. The highest scoring alignment is kept, and aligned reads are chosen if their score is higher than the maximal pairwise difference in scores. Regardless of data type, we do not include GCG sites because their status is biologically ambiguous. Thus, we denote GC sites that are not followed by a G as GCH and CG sites that are not preceded by a G as HCG.

After the preprocessing is complete, we assign all sites a numeric value. GCH sites that are methylated are assigned the value -4; unmethylated are assigned -1. Bases between two methylated GCH sites are assigned -3, and those between two unmethylated GCH sites are assigned -2. These in-between bases represent what we refer to as a methylation ‘patch’. If two consecutive GCH sites do not have the same methylation state, the bases in between are assigned the value -2.5 resulting in a gray color. The same scheme with positive values is used for HCG sites. A white patch is due to missing data, especially for sparse single-cell data in which the methylation status of an HCG or GCH site is unknown due to missed coverage in sequencing. A light gray border is inserted from the most external HCG and GCH sites to the end of the aligned sequence. This unique assignment provides a structure with which the data can be ordered using numerical methods and then visualized.

**References**

Xi, Y., Li, W. BSMAP: whole genome bisulfite sequence MAPping program. BMC Bioinformatics 10, 232 (2009). https://doi.org/10.1186/1471-2105-10-232

**2. Description of methylscaper ordering procedure**

The primary ordering method used by methylscaper is derived from a weighted principal components analysis. Let *X* denote the *n* x 2*b* representational matrix, where *n* denotes the number of molecules or cells and *b* denotes the number of base pairs (*X* is thus formed by joining the columns of the GCH and HCG matrices row-wise). The weight of row *i*, denoted *w_i_,* is computed as the number of bases in methylated patches that lie within a region of the molecules indicated by the user. Numerically, we compute *w_i_* as

*w_i_* = $\sum_{j=1}^{2b} I(X_{i,j})$

where *I* is an indicator function, equal to 1 if *X_i,j_* falls within a methylation patch of interest, and equal to 0 otherwise. We then normalize all of the weights so that they sum to 1 and multiply each row of *X* by the square-root of its normalized weight, forming the weighted representational matrix *X^*^*. We then compute the Singular Value Decomposition of *X^*^*, i.e.,

*USV^T^* = *X^*^*

The order of the entries in the first column of *U* is then used as the order of the molecules in generating the methylscaper plots for both HCG and GCH methylation status.

**3. Computational performance**

Methylscaper’s runtime performance was evaluated using the *microbenchmark* R package. We compared the principal component analysis (PCA) method with the hierarchical clustering (HC) method provided by the *seriation* package by running these methods on the example MAPit-BGS data. To evaluate the performance of each method on larger scale data, we replicated the rows of the representational matrix 2, 5, and 10 times to increase the total number of molecules. Methylscaper scales to analyzing large datasets while illuminating heterogeneous epigenetic features that will be useful as single-cell approaches continue to evolve. Summary statistics after 100 runs of each method are given below in Supplementary Table S1. All times are given in milliseconds.

**Supplementary Table S1**

| Method | Dataset | Min  (milliseconds) | Mean  (milliseconds) | Max  (milliseconds) |
| --- | --- | --- | --- | --- |
| HC | 1x | 218 | 233 | 527 |
| HC | 2x | 423 | 445 | 696 |
| HC | 5x | 1207 | 1243 | 1588 |
| HC | 10x | 2898 | 3176 | 3805 |
| PCA | 1x | 247 | 265 | 549 |
| PCA | 2x | 474 | 493 | 812 |
| PCA | 5x | 1322 | 1399 | 1741 |
| PCA | 10x | 3182 | 3371 | 3829 |

**4. Additional details on analysis of case-study datasets**

i) MAPit-BGS: The raw data is included in Supplementary Data. The file reference.fa is the sequence of the promoter region of the *EMP2AIP1* gene. The file seq_file.fasta is the sequences of all reads generated by the MAPit-BGS experiment. After quality control in the bioinformatic pipeline, there were 149 high-quality, single-molecule reads. We ran methylscaper with weighting on the HCG features from position 308 to 475, we then iteratively refined molecules from 1-54, 1-39, and 1-24. All plots were made with options plotFast=FALSE and drawKey=FALSE.

ii) scNMT-seq: We downloaded the scNMT-seq data from GSE109262, which contained 61 cells that passed quality control in the original publication in Clark et al. 2018. The data were originally aligned using GRCm38. We focused on a region of the *Eefg1* gene from (TSS-200, TSS+500), where the TSS is located on chromosome 19 at 8,967,041 bp. We ran methylscaper with weight on the GCH features from base 47 to 358 and refined molecules from 27 to 42.

**Supplementary Figures**

**
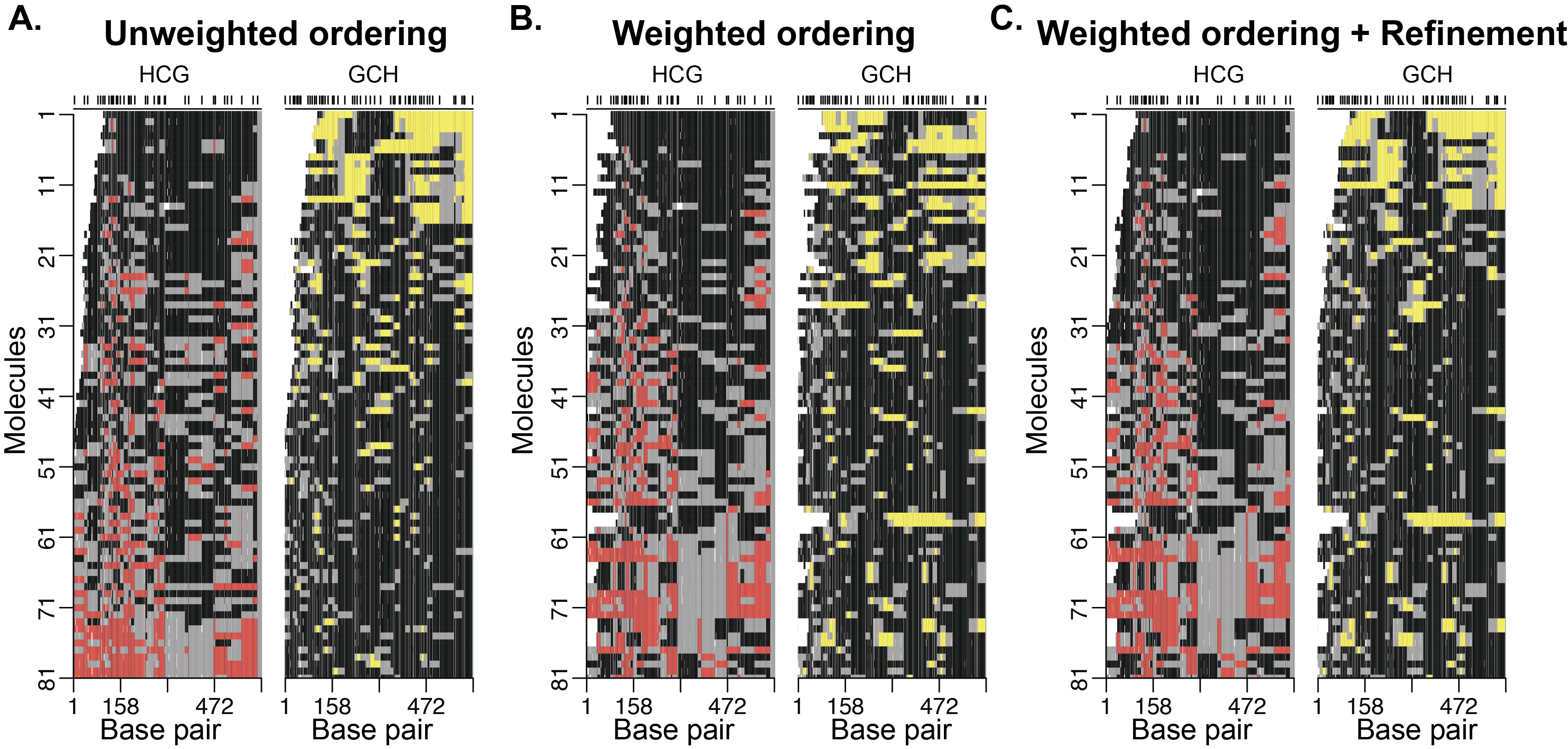
**

Supplementary Figure 1: A comparison of ordering procedures provided by methylscaper using molecules 20-100 from Figure 1 as an example. A. methylscaper plot ordered using the unweighted PCA. B. methylscaper plot of data ordering with a weighted PCA where ordering is focused on the red patch from 308 bp to 475 bp. C. The weighted methylscaper plot with weighting as in (B) and with refinement ordering on the first 40 molecules. Now, both the specific regions with accumulated HCG as well as patches of accessible and hence methylated GCH at the TSS in a subset of molecules are clearly visible.

Supplementary Figure 2: Scatter plots of the number of methylated sites *versus* the first principal component of the representational state matrix. A. Number of endogenously methylated sites *versus* PC1 values. B. Number of accessible sites *versus* PC1 values.


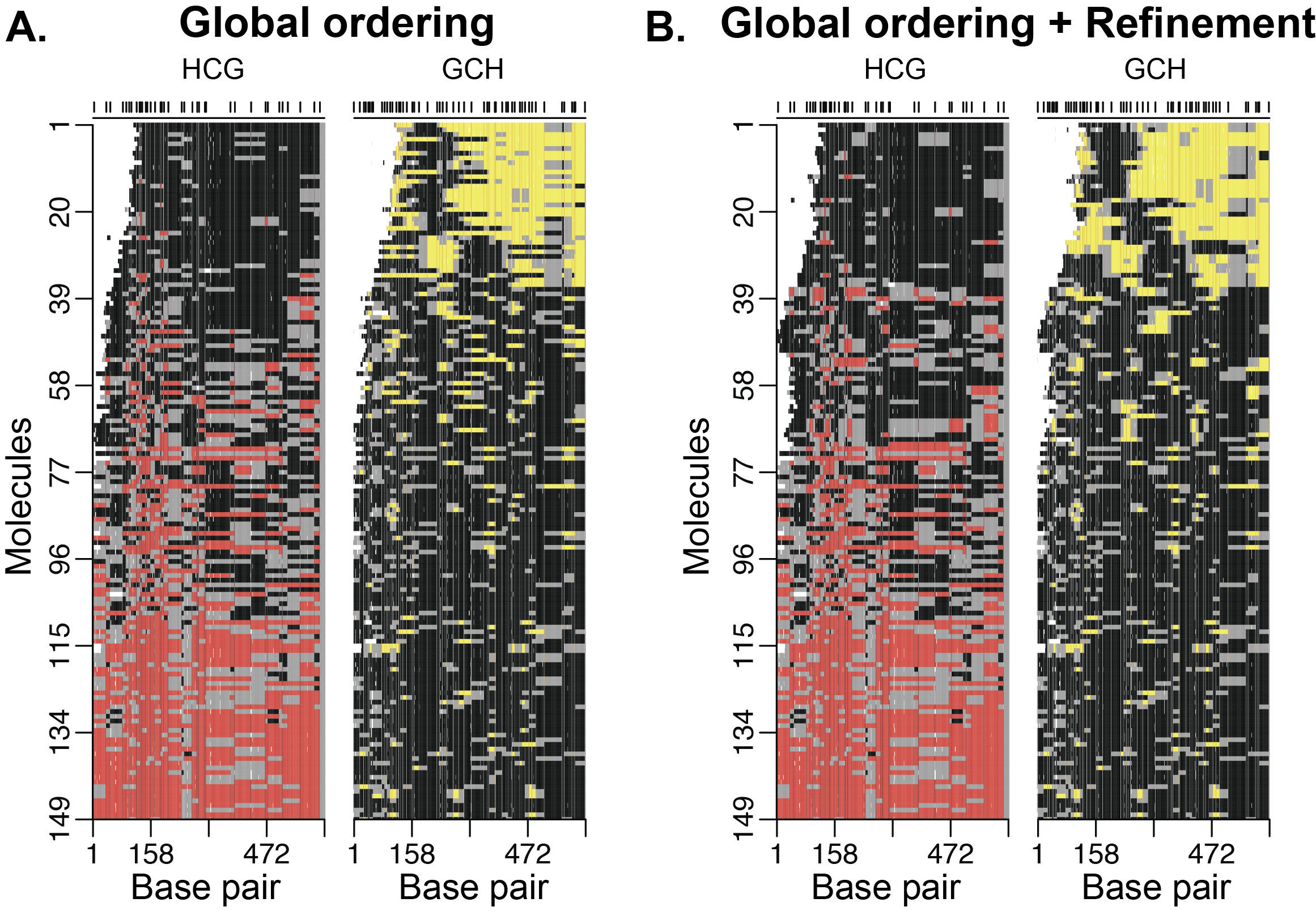


Supplementary Figure 3: A comparison of unrefined and refined methylscaper plots. A. methylscaper plot after the initial global ordering by PCA. B. The methylscaper plot after refining the ordering of a subset of the first 70 molecules, followed by refinement of the first 35 molecules.

Supplementary Figure 4: Experiment-wide summary plots generated by methylscaper. A. The percentage of molecules methylated at each HCG or GCH site. B. A histogram of the proportion of HCG sites that are methylated within individual molecules. C. A histogram of the proportion of GCH sites that are methylated within individual molecules.


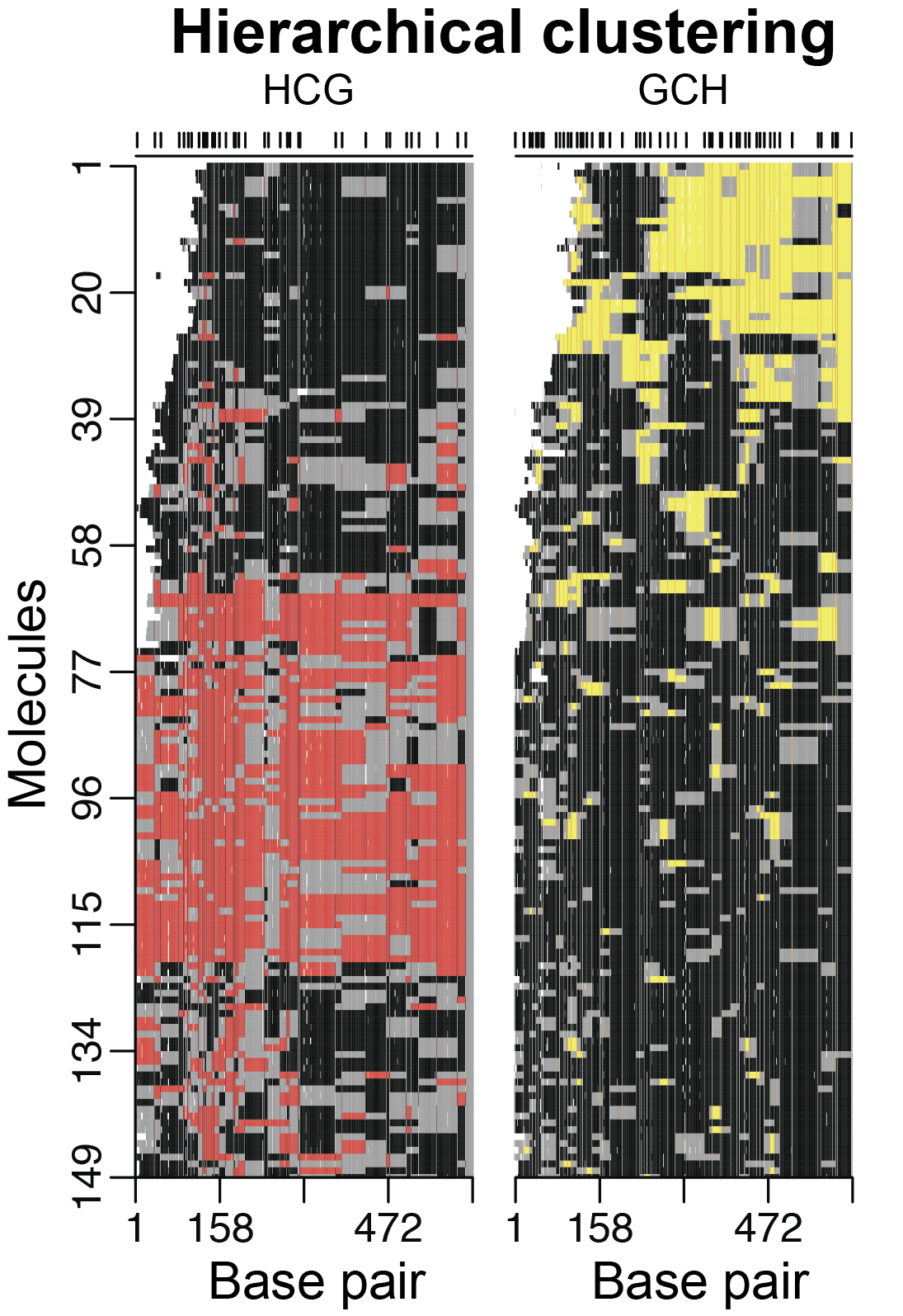


Supplementary Figure 5: A methylscaper plot with the molecule ordering computed with hierarchical clustering.

Supplementary Figure 6. Examples of alternative plots used to visualize methylation data. (A) A line plot of the moving average (50 bp window) of methylation and accessibility across all molecules. (B) A lollipop plot generated from the NOMePlot software with a subset of 28 molecules.


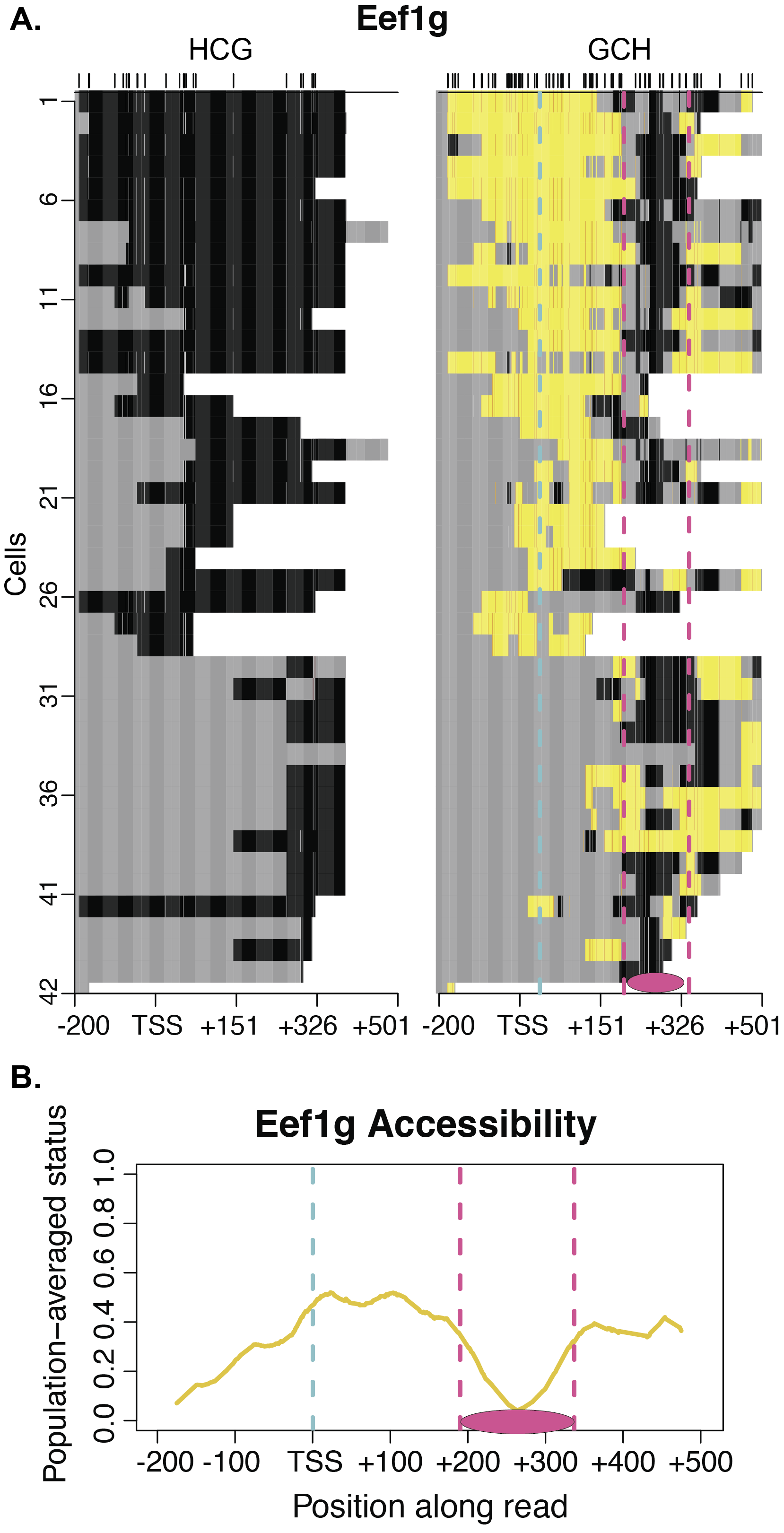
Supplementary Figure 7: Analysis of the *Eef1g* gene from a scNMT-seq dataset. A. Methylscaper plot of *Eef1g* surrounding the transcription start site (TSS). The region immediately near the TSS (+/- 200 bp) is highly accessible, while a nucleosome downstream of the TSS is centered around +250 bp. B. A moving average (50 bp window) of accessibility across all molecules also indicates evidence of a well-positioned +1 nucleosome.
